# Impact of Activation Conditions on the Electrochemical Performance of Rice Straw Biochar for Supercapacitor Electrodes

**DOI:** 10.3390/molecules30030632

**Published:** 2025-01-31

**Authors:** Jialuo Cheng, Yumeng Lu, Ya Sun, Sunhua Deng, Heng Yang, Manman Zhang, Chunlei Wang, Juntao Yan

**Affiliations:** 1 Key Laboratory of Agricultural Waste Resource Utilization in Hubei Province, College of Chemistry and Environmental Engineering, Wuhan Polytechnic University, Wuhan 430023, China; 15972060421@163.com (J.C.); yhxg666@sina.com (H.Y.); zhangmm@hust.edu.cn (M.Z.); wangcl@whpu.edu.cn (C.W.); 2 Institute of Plant Protection and Soil Fertilizer, Hubei Academy of Agricultural Sciences, Wuhan 430064, China; 3 School of Nursing and Health Management, Wuhan Donghu University, Wuhan 430212, China; sunya230@163.com; 4 College of Construction Engineering, Jilin University, Changchun 130021, China; dengsh@jlu.edu.cn

**Keywords:** rice straw biochar, activation, electrodes, supercapacitor

## Abstract

Biochar, derived from agricultural waste, has gained significant attention as a sustainable material for energy storage applications due to its high surface area, tunable porosity, and environmental benefits. This study investigates the electrochemical performance of rice straw biochar (RSBC) as an electrode material, with a focus on the effects of activation temperature, activation ratio, and activation time. Among the prepared samples, RSBC-2, activated at 800 °C with a 1:2 KOH ratio for 0.5 h, exhibited the best electrochemical performance. Characterization of RSBC and RSBC-2 showed significant improvements in surface area and pore structure. Specifically, the BET surface area of RSBC-2 increased to 939.40 m^2^ g^−1^, with a reduced average pore size of 2.27 nm. Electrochemical testing revealed that RSBC-2 achieved specific capacitances of 296, 281, 272, 260, and 240 F g^−1^ at current densities of 0.2, 0.5, 1, 2, and 5 A g^−1^, respectively, with a capacity retention rate of 81%. The improved electrochemical performance of RSBC-2 is attributed to its larger surface area and enhanced pore structure, which facilitate better charge storage and overall electrochemical behavior, making it a promising candidate for energy storage applications.

## 1. Introduction

Supercapacitors are essential energy storage devices renowned for their high-power density, rapid charge/discharge rates, and long cycle life, making them ideal for applications such as electric vehicles and renewable energy systems [1,2]. However, their relatively low energy density compared to conventional batteries restricts their use in certain energy storage applications [3,4]. This constraint arises primarily from the limited charge storage capacity of supercapacitors, which is largely determined by the surface area, pore structure, and electrical conductivity of the electrode materials [5].

A key challenge in supercapacitor development is enhancing their energy density. Although supercapacitors are efficient at providing rapid energy bursts, their energy storage capacity falls short for applications that demand continuous power, such as grid storage and electric vehicles. This limitation is primarily due to the low charge storage capacity of conventional electrode materials, which rely on physical ion adsorption. To address this, research has focused on developing materials with increased surface area and optimized pore structures to enhance ion storage and improve performance [6,7].

Another key challenge is the cost and sustainability of electrode materials. Carbon-based materials [8,9], including activated carbon [10], graphene [11], and carbon nanotubes [12], have been extensively studied as electrode materials due to their high surface area, excellent electrical conductivity, and structural stability. Among these, activated carbon remains the most commonly used material in commercial supercapacitors because of its favorable balance of cost, performance, and environmental sustainability [13,14]. Traditional activated carbon production typically relies on expensive and nonrenewable feedstocks such as wood, coal, petroleum residues, peat, lignite, and polymers [15]. In recent years, there has been increasing interest in using biochar—a carbon-rich material derived from the pyrolysis of biomass—as a promising alternative precursor for activated carbon [14]. Biochar, often produced from agricultural residues such as rice straw [16], rice husk [17], and corn husk [18], offers several advantages over conventional activated carbon, including sustainability, low cost, and the potential for large-scale production from renewable resources [19,20]. Biochar-based electrodes enhance the sustainability of supercapacitors by utilizing abundant, renewable agricultural waste, while also providing an environmentally friendly alternative to fossil-based materials. With its unique porous structure, high surface area, and functional groups, biochar is an ideal candidate to improve electrochemical performance, making supercapacitors more cost-effective and environmentally sustainable without compromising performance [15].

Chemical activation is a widely used method to enhance the properties of biochar. Acidic, alkaline, and oxidation treatments are commonly applied to activate biochar, utilizing acidic agents such as HCl, HNO_2_, H_2_SO_4_, and H_3_PO_4_, alkaline agents like KOH, NaOH, and K_2_CO_2_, and oxidizing agents such as H_2_O_2_ and KMnO_4_ [21]. Mahmoud et al. [22] demonstrated that HCl activation of kenaf fiber biochar significantly increased its BET surface area, resulting in the formation of honeycomb-shaped pores of varying sizes. Acid treatment improves the pore structure of biochar by enhancing surface area and porosity, likely due to the removal of surface impurities. Furthermore, it introduces or increases functional groups such as amino, carboxylic, and other oxygen-containing groups [23]. Alkali treatment also improves biochar properties by enhancing both its pore structure and surface functional groups [24]. Dehkhoda et al. [25] showed that KOH-activated biochar with a microporous structure exhibited high capacitance (222–245 F g^−1^), making it suitable for water treatment and energy storage applications. The introduction of mesopores, while slightly reducing capacitance (182–240 F g^−1^), improved electrode resistance and capacitive behavior. However, the relationship between activation conditions of rice straw biochar and its electrochemical performance remains an area requiring further investigation.

Herein, rice straw was selected as the precursor for biochar, which was produced through thermal treatment and KOH activation. The effects of activation temperature, activation ratio, and activation time on the crystallinity, morphology, and electrochemical performance of the activated rice straw biochar, particularly for supercapacitor electrodes, were systematically investigated and discussed.

## 2. Results and Discussions

### 2.1. Effects of Activation Temperature

During the synthesis of biochar, the activation temperature plays a crucial role in determining the pore structure, purification level, chemical properties, and thermal stability of the biochar [26]. At lower temperatures, pore opening is limited, while higher temperatures may cause structural damage. Therefore, the activation temperatures for rice straw biochar were controlled at 700 °C, 800 °C, and 900 °C under constant conditions, with the resulting samples designated as RSBC-700, RSBC-800, and RSBC-900.

**Figure 1:**
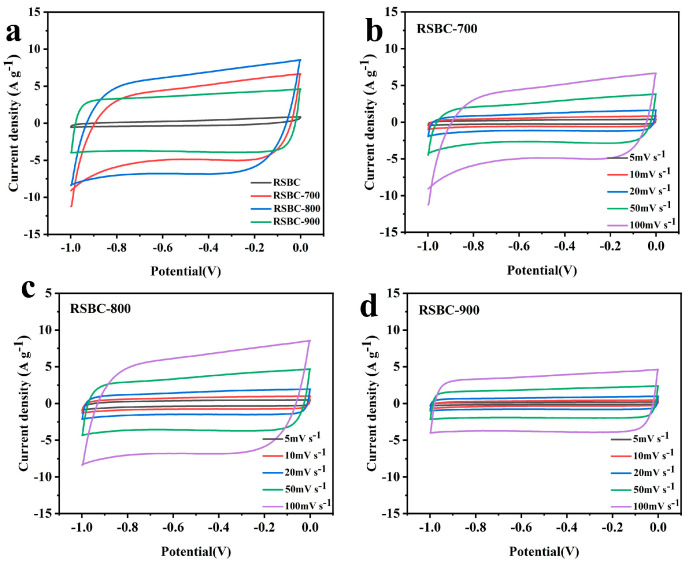
Cyclic voltammetry curves of rice straw biochar at different activation temperatures with a scan rate of 100 mV s^−1^ (**a**) and under various scan rates (**b**–**d**).

Figure 1a displays the cyclic voltammetry (CV) curves of rice straw biochar electrodes activated at different temperatures, measured at a scan rate of 100 mV s^−1^. As shown in Figure 1a, all activated electrode materials exhibit a well-defined, nearly rectangular shape, which indicates excellent double-layer capacitance properties. In comparison, the CV curve of the pristine RSBC sample has a smaller area, reflecting its lower charge storage capacity. Among the three activation temperatures, the sample activated at 800 °C demonstrates the best shape and the largest area, suggesting that it may have superior charge storage capabilities. Figure 1b–d further illustrates that, when the scan rate increases from 5 to 100 mV s^−1^, the CV curves of rice straw biochar electrodes activated at different temperatures maintain their rectangular shape. This indicates that the activated electrode materials exhibit a fast electrochemical response and good cycling stability, consistent with the typical CV curve shapes observed in electric double-layer capacitor (EDLC) supercapacitors.

**Figure 2:**
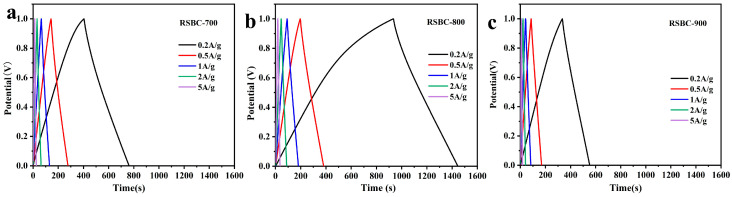
Galvanostatic charge–discharge curves of rice straw biochar at different activation temperatures under various current densities.

**Figure 3:**
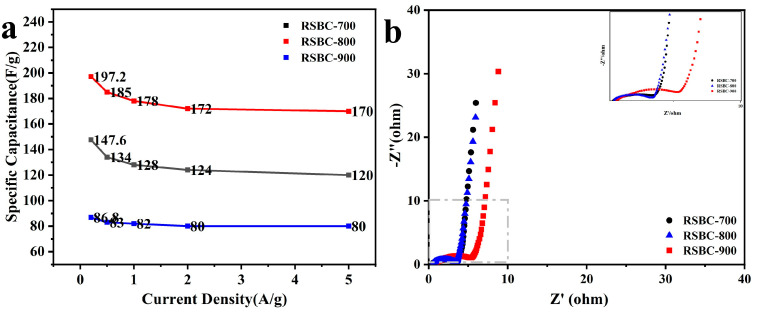
Specific capacitance at different current densities (**a**) and Nyquist plots (**b**) of rice straw biochar at different activation temperatures.

The galvanostatic charge–discharge (GCD) tests of rice straw biochar at different activation temperatures were conducted under current densities ranging from 0.2 to 5 A g^−1^, with a voltage range of 0–1 V; the results are shown in Figure 2. As observed in Figure 3, the sample activated at 800 °C exhibits the steepest slope and the highest capacitance, indicating superior electrochemical performance. With increasing current density, the discharge rate of the electrode material accelerates, leading to a reduction in discharge time. Additionally, when the current density increases from 0.2 A g^−1^ to 5 A g^−1^, the GCD curves of all samples exhibit an isosceles-triangle-like shape, signifying good reversibility of the biochar samples. However, as the current density increases, the area under the GCD curve decreases, leading to a reduction in the corresponding capacitance. The specific capacitance values of the activated rice straw biochar were calculated based on the GCD results, and the data are summarized in Table 1. From the table, it can be seen that the specific capacitance of rice straw biochar increases with activation temperature at various current densities. At an activation temperature of 800 °C, the rice straw biochar exhibits the highest specific capacitance across all current densities. The specific capacitance of RSBC-800 at current densities of 0.2, 0.5, 1, 2, and 5 A g^−1^ are 197.2, 185, 178, 172, and 170 F g^−1^, respectively.

Figure 3a shows the specific capacitance at different current densities for rice-straw-based carbons activated at various temperatures. From the figure, it is evident that, as the activation temperature increases from 700 °C to 800 °C, the specific capacitance at different current densities improves. However, when the activation temperature is further raised to 900 °C, the specific capacitance decreases instead of continuing to increase. At an activation temperature of 800 °C, RSBC-800 exhibits a specific capacitance retention of 81.3% across current densities ranging from 0.2 to 5 A g^−1^. Generally, higher activation temperatures promote molecular and atomic movement within the material, which enhances the electrochemical activity and specific surface area of the electrode material, thereby increasing the specific capacitance. However, excessively high activation temperatures may cause structural degradation of the electrode material, as elevated temperatures can induce pyrolysis or oxidation reactions that diminish the active sites, ultimately reducing the stability and specific capacitance of the material.

**Figure 4:**
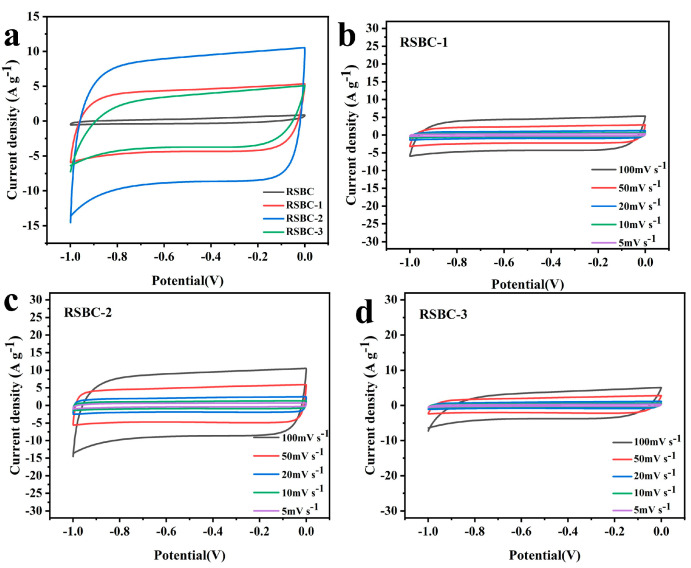
Cyclic voltammetry curves of rice straw biochar with different activation ratios with a scan rate of 100 mV s^−1^ (**a**) and under various scan rates (**b**–**d**).

Figure 3b presents the Nyquist plots for rice straw biochar at different activation temperatures. In the mid-to-high frequency region, the diameter of the semicircle on the real axis represents the charge transfer resistance (Rct). In the high-frequency range, the distance from the first intersection of the curve with the real axis to the center of the co-ordinate axis corresponds to the equivalent series resistance (Rs), which includes the contact resistance between the electrode material and the electrolyte, the contact resistance between the electrode and the foam nickel current collector, and the interface resistance between the electrode and the electrolyte [27]. From Figure 4b, it can be observed that the series resistances (Rs) for RSBC-700, RSBC-800, and RSBC-900 are 0.41, 0.50, and 0.73 Ω, respectively. The charge transfer resistances (Rct) for these samples are 3.41, 3.00, and 4.79 Ω, respectively. RSBC-800 demonstrates the lowest Rct, indicating superior conductivity and efficient electron transfer.

### 2.2. Effects of Activation Ratio

The activation ratio is crucial for determining the porosity, surface area, and structural integrity of biochar. While a higher ratio enhances adsorption capacity and suitability for energy storage, an overly high ratio can compromise the structure of material [28]. Therefore, optimizing this ratio is essential for achieving a balance between porosity and structural stability. To investigate this effect and keeping other conditions constant, the ratio of rice straw biochar to KOH was adjusted to 1:1, 1:2, and 1:3, with the resulting samples being labeled as RSBC-1, RSBC-2, and RSBC-3, respectively.

Figure 4a shows the CV curves of RSBC electrodes at different activation ratios, measured at a scan rate of 100 mV s^−1^. As observed in the figure, the activation ratio significantly enhances the electrochemical performance compared to the unactivated RSBC. When the activation ratio of rice straw biochar to KOH increases from 1:1 to 1:2, both the rectangular shape and the area of the CV curve show significant improvement, indicating enhanced electrochemical properties. However, further increasing the activation ratio to 1:3 leads to a decline in electrochemical performance, suggesting that an excessively high activation ratio may negatively impact the material’s structure or electrochemical behavior. Figure 4b–d display the CV curves of RSBC at different activation ratios, measured at various scan rates. It can be observed that, as the scan rate increases from 5 mV s^−1^ to 100 mV s^−1^, the CV curves of rice straw biochar electrodes with different activation ratios maintain a similar rectangular shape. This indicates that, even at higher scan rates, the electrodes retain their good electrochemical reversibility and capacitive behavior, further demonstrating the favorable electrochemical performance of RSBC at different activation ratios.

**Figure 5:**
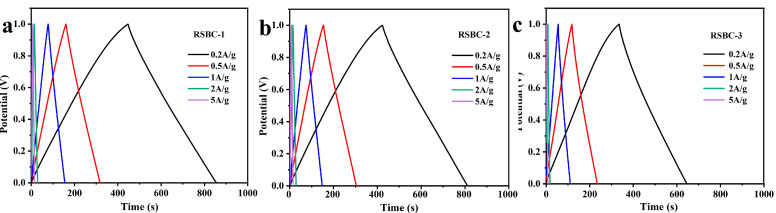
Galvanostatic charge–discharge curves of rice straw biochar with different activation ratios under various current densities, (**a**) RSBC-1, (**b**) RSBC-2, and (**c**) RSBC-3.

The galvanostatic charge–discharge (GCD) tests of rice straw biochar with different activation ratios were conducted at current densities ranging from 0.2 A g^−1^ to 5 A g^−1^, within a voltage window of 0 to 1 V, and the results are shown in Figure 5. From the charge–discharge curves, it can be observed that, when the ratio of RSBC to KOH is 1:2, the electrode sample exhibits the largest triangular area, the steepest slope, and the highest capacitance, indicating superior electrochemical performance. The specific capacitances of RSBC at different activation ratios and current densities were calculated from the GCD curves, and the results are summarized in Table 2. As shown in the table, when the ratio of RSBC to KOH is 1:2, the specific capacitance at various current densities is optimal. RSBC-2 demonstrates specific capacitances of 296, 281, 272, 260, and 240 F g^−1^ at current densities of 0.2, 0.5, 1, 2, and 5 A g^−1^, respectively, indicating the best overall electrochemical performance among the samples.

**Figure 6:**
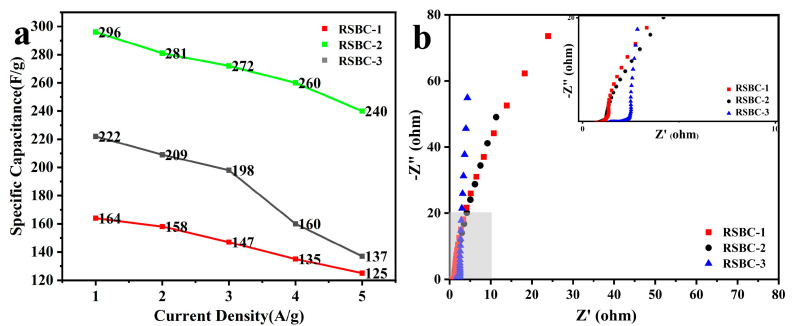
Specific capacitance at different current densities (**a**) and Nyquist plots (**b**) of rice straw biochar with different activation ratios.

Figure 6a presents the specific capacitance versus current density curve for rice straw biochar (RSBC) with different activation ratios. It is evident that RSBC-2 exhibits a significant improvement compared to both RSBC-1 and RSBC-3. Specifically, RSBC-2 demonstrates nearly a 50% increase in specific capacitance over RSBC-1 across the entire range from 0.2 A g^−1^ to 5 A g^−1^ and nearly a 100% increase compared to RSBC-3. The capacitance retention rates of RSBC-1, RSBC-2, and RSBC-3 at current densities from 0.2 A g^−1^ to 5 A g^−1^ are 61.7%, 81%, and 76.2%, respectively. This suggests that an optimized activation ratio increases the content of active sites in the electrode material, thereby enhancing its specific capacitance. However, an excessively high activation ratio can lead to an overaccumulation of active sites, which may destabilize the electrode material and reduce its specific capacitance. Figure 6b compares the electrochemical impedance of RSBC with different activation ratios. From the figure, it is clear that the series resistance (Rs) values for RSBC-1, RSBC-2, and RSBC-3 are 0.57, 0.39, and 1.30 Ω, respectively. Additionally, the charge transfer resistances (Rct) are 0.8, 0.77, and 1.18 Ω, respectively. It is evident that RSBC-2 exhibits the lowest charge transfer resistance (Rct), indicating superior electrochemical performance and more efficient charge transfer compared to the other samples.

### 2.3. Effects of Activation Time

Activation time plays a significant role in determining the pore structure, surface chemical properties, and conductivity of biochar [29], which, in turn, influences its capacitance performance. To investigate this effect and keeping other conditions constant, activation times of 0.5 h, 6 h, and 12 h were selected. The corresponding samples were named RSBC-0.5 h, RSBC-6h, and RSBC-12 h, respectively.

**Figure 7:**
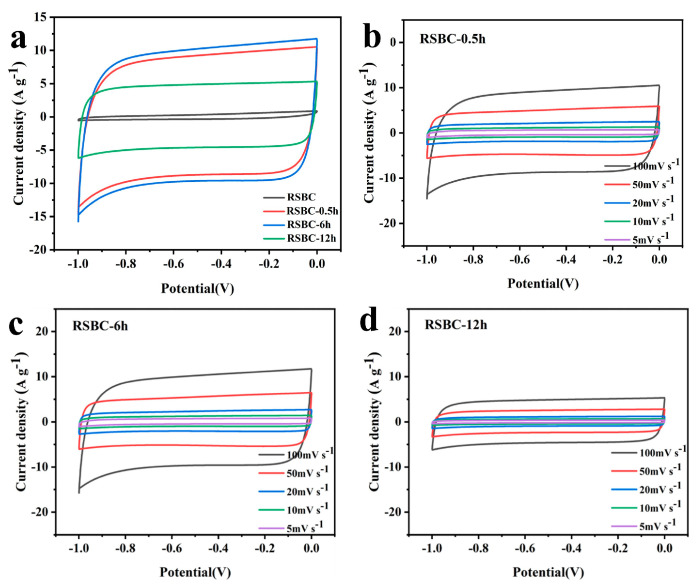
Cyclic voltammetry curves of rice straw biochar with different activation times with a scan rate of 100 mV s^−1^ (**a**) and under various scan rates (**b**–**d**).

Figure 7a shows the CV curves of rice straw biochar electrodes with different activation times at a scan rate of 100 mV s^−1^. It can be observed that the areas of the CV curves for RSBC-0.5 h and RSBC-6 h are significantly larger than that of RSBC-12 h, indicating that an appropriate activation time enhances the electrochemical performance of the electrode material. However, an excessively long activation time, as seen in RSBC-12 h, leads to negative effects such as over-dissolution and crystal agglomeration, which ultimately degrade the electrochemical performance. Figure 7b–d present the CV curves of rice straw biochar at different activation times under varying scan rates. As the scan rate increases from 5 mV s^−1^ to 100 mV s^−1^, the CV curves for all samples retain a nearly rectangular shape, indicating good capacitive behavior. The electrochemical performance of RSBC-0.5 h and RSBC-6 h is nearly identical, while RSBC-12 h exhibits a significantly smaller rectangular area. This suggests that the electrochemical performance of RSBC-12 h is inferior to that of RSBC-0.5 h and RSBC-6 h, further supporting the idea that an optimal activation time is crucial for achieving high electrochemical performance.

**Figure 8:**
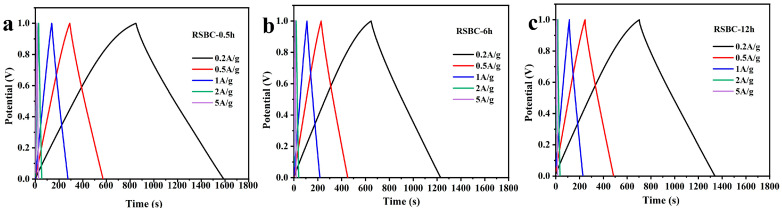
Galvanostatic charge–discharge curves of rice straw biochar with different activation times under various current densities, (**a**) RSBC-0.5 h, (**b**) RSBC-6 h, and (**c**) RSBC-12 h.

The galvanostatic charge–discharge (GCD) tests of rice straw biochar at different activation times were conducted under current densities ranging from 0.2 to 5 A/g, with a voltage range of 0–1 V, and the results are shown in Figure 8. Based on the charge–discharge curves in the figure, it can be observed that the electrochemical performance is best when the activation time of RSBC with KOH is 0.5 h. The specific capacitance of biochar with different activation times at various current densities was calculated from the GCD curves, and the results are summarized in Table 3. As shown in the table, the specific capacitance of rice straw biochar decreases with increasing activation time at all current densities. Specifically, when the activation time is 0.5 h, the rice straw biochar exhibits the highest specific capacitance across all current densities. For RSBC-0.5 h, the specific capacitance at 0.2, 0.5, 1, 2, and 5 A g^−1^ is 296, 281, 272, 260, and 240 F g^−1^, respectively. This confirms that shorter activation times are more beneficial for optimizing the electrochemical performance of rice straw biochar.

**Figure 9:**
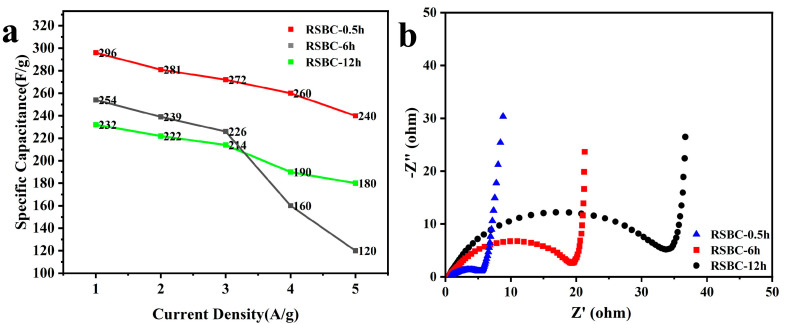
Specific capacitance at different current densities (**a**) and Nyquist plots (**b**) of rice straw biochar with different activation times.

Figure 9a shows the specific capacitance versus current density curves for rice straw biochar with different activation times. It is clearly observed that RSBC-0.5 h outperforms both RSBC-6 h and RSBC-12 h, especially at higher current densities of 2 A g^−1^ and 5 A g^−1^, where the capacity retention rate exhibits a significant improvement. The capacity retention rates for RSBC-0.5 h, RSBC-6 h, and RSBC-12 h at current densities ranging from 0.2 A g^−1^ to 5 A g^−1^ are 81%, 47.2%, and 77.6%, respectively. This indicates that, with an activation time of 0.5 h, the active materials are well dispersed and fully utilized in the electrode material, leading to optimal effective utilization. Furthermore, the shorter activation time minimizes the dissolution of the electrode material, thereby enhancing its cycling stability. Figure 9b presents a comparison of electrochemical impedance spectroscopy (EIS) for rice straw biochar with different activation times. It is evident from the figure that the series resistance (Rs) values for RSBC-0.5 h, RSBC-6 h, and RSBC-12 h are 0.42, 0.35, and 0.33 Ω, respectively. Meanwhile, the charge transfer resistance (Rct) values are 5.16, 18.85, and 33.47 Ω, respectively. These results suggest that RSBC-0.5 h has the lowest charge transfer resistance, which corresponds to the highest specific capacitance observed in its galvanostatic charge–discharge (GCD) measurements. This indicates that a shorter activation time significantly improves the electrochemical performance of the rice straw biochar. The cycling stability tests for RSBC-2 are shown in Figure S1. The RSBC-2 was cycled for 1000 cycles at a current density of 5 A g^−1^. After 1000 cycles, RSBC-2 retained 100% of its capacity, indicating that the electrode materials exhibit excellent cycling stability.

### 2.4. Morphological and Structural Characterization of Rice Straw Biochar Before and After Activation

According to the electrochemical performances of rice straw biochar under different activation conditions, it can be found that the RSBC-2, prepared by a 1:2 KOH-to-biochar ratio, activated for 0.5 h at 800 °C, showed the best electrochemical performance. Therefore, RSBC-2 and nonactivated RSBC have been compared to investigate the structural and morphological differences before and after activation.

**Figure 10:**
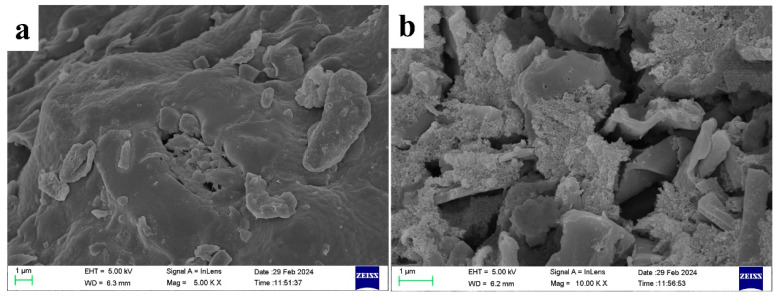
SEM images of RSBC (**a**) and RSBC-2 (**b**).

Figure 10 presents the scanning electron microscope (SEM) images of rice straw biochar before and after activation. As shown in Figure 10a, the surface of RSBC is rough and characterized by large, block-like structures. After activation (Figure 10b), RSBC-2 exhibits a looser particle morphology with some degree of agglomeration. This structural change is likely due to the dissolution and removal of carbon during KOH activation, as well as the release of gases throughout the process [30]. These factors contribute to the formation of voids and pores both on the surface and within the biochar, altering its overall morphology.

**Figure 11:**
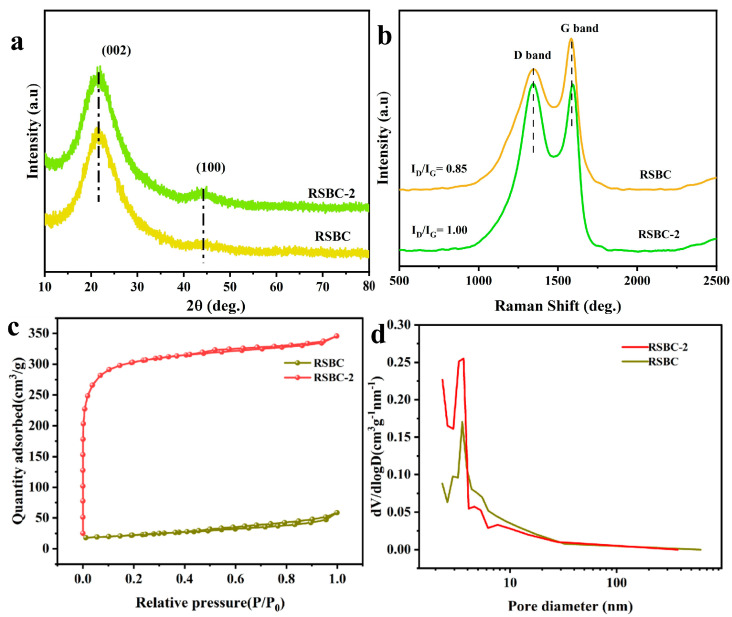
XRD patterns (**a**), Raman spectra (**b**), nitrogen adsorption–desorption isotherms (**c**), and pore size distribution (**d**) of RSBC and RSBC-2.

Figure 11a shows the XRD patterns of rice straw biochar before and after activation. As seen in the figure, the characteristic peaks of RSBC and RSBC-2 appear at 2θ = 21.5° and 44.3° [31,32]. After activation, the diffraction peaks of RSBC-2 remain nearly identical to those of RSBC, indicating that KOH activation has a minimal impact on the overall structure of the rice straw biochar. The two broad peaks at 21.5° and 44.3° for both RSBC and RSBC-2 correspond to the (002) and (100) crystallographic planes of carbon, respectively. Notably, after KOH activation, the intensity of the (101) crystallographic plane increases.

The Raman spectra typically exhibit two characteristic peaks: the D band at 1350 cm^−1^, which is associated with defects or disorder in the graphene-like structure, and the G band around 1580 cm^−1^, corresponding to highly ordered graphite structures. The intensity ratio, R (I_D_/I_G_), between the D and G bands reflects the structural integrity, with a higher ratio indicating more disorder and defects [33]. The Raman spectra of RSBC and RSBC-2 (Figure 11b) show minimal differences, suggesting similar structural ordering. The R values for RSBC and RSBC-2 are 0.85 and 1.00, respectively. After activation, the R value increases, which can be attributed to the defect sites generated during KOH activation.

Figure 11c,d shows the nitrogen adsorption–desorption isotherms and pore size distribution of rice straw biochar before and after activation. As seen in the figure, RSBC exhibits a typical Type II isotherm, while RSBC-2 displays a typical Type I isotherm with an H3 hysteresis loop. In the low-pressure range (P/P₀ < 0.4), the isotherm of RSBC-2 shows a sharp increase, indicating the presence of abundant micropores in the material. Since electrochemical reactions primarily occur within micropores, the high density of micropores in RSBC-2 significantly enhances its electrochemical performance. As the relative pressure (P/P₀) exceeds 0.9, the curve exhibits a slight increase, suggesting the presence of macropores in RSBC-2. The specific surface area and pore size of RSBC and RSBC-2 are summarized in Table 4. The specific surface areas of RSBC and RSBC-2 are 75.34 and 939.40 m^2^·g^−1^, while their total pore volumes are 0.09 and 0.53 cm^3^·g^−1^, respectively. After activation, both the specific surface area and total pore volume increase, indicating that activation generates voids and pores within the rice straw biochar.

TG-DTG curves of rice straw biochar before and after activation were constructed. To investigate the thermal stability of different samples, the initial sample RSBC and the best-performing RSBC-2 were subjected to thermal stability tests at a heating rate of 10 °C/min in a nitrogen atmosphere. As shown in Figure S2, the TG-DTG comparison of rice straw biochar before and after activation is presented. The TG curve shows the change in sample mass with respect to temperature and time, while the DTG curve reflects the rate of mass change with respect to temperature and time. Figure S2a shows the TG and DTG curves of RSBC, where the pyrolysis can be divided into three stages. The first stage occurs from 0 °C to approximately 150 °C, during which a mass loss of about 12% is observed, primarily due to the surface-adsorbed moisture and some volatile organic compounds. The second stage occurs from 150 °C to 600 °C, with a mass loss of about 20%. The third stage, from 600 °C to 800 °C, shows a mass loss of approximately 40%. At higher temperatures, the main components of RSBC undergo pyrolysis and mass loss, which leads to multiple peaks in mass loss. Figure S2b shows the TG and DTG curves of RSBC-2, where the mass loss is divided into two stages. The first stage occurs from 0 °C to around 300 °C, with a mass loss of about 10%, and the second stage occurs from 300 °C to 800 °C, with a mass loss of about 60%. Figure S2c,d provide a clearer observation of the pyrolysis rate and mass loss proportion of RSBC and RSBC-2. In the first stage, the higher the surface moisture content, the faster the rate of mass loss and the greater the proportion of mass loss. For energy storage materials, higher moisture content can lead to oxidation and corrosion of the electrode material, which, in turn, reduces its electrochemical performance.

KOH activation enhances the electrochemical performance of biochar through several mechanisms. First, it promotes pore formation and increases surface area by reacting with the carbon structure to create potassium-based compounds, which decompose the carbon matrix and generate microporosity. This significantly improves the material’s porosity, crucial for electrochemical performance. Second, KOH activation removes oxygenated functional groups, such as hydroxyl, carboxyl, and carbonyl groups, which can hinder conductivity and performance, thus improving both conductivity and stability [34]. Third, the activation process increases the graphitization degree of biochar, enhancing electrical conductivity and charge/discharge rates. Finally, potassium ions introduced during activation can contribute to ion storage, improving specific capacitance; however, excessive potassium incorporation may cause structural degradation, underscoring the need for optimization of activation parameters [35].

## 3. Experimental

### 3.1. Materials Preparation

Rice straw sourced from Hubei Province, China, was initially cleaned, dried, and ground into a fine powder using a milling machine. The resulting powder was passed through a 100-mesh sieve to separate finer particles of rice straw.

A total of 10 g of the dried rice straw powder was weighed and placed into a crucible. The crucible was then transferred to a tube furnace, where the temperature was gradually increased to 600 °C at a rate of 5 °C/min under a nitrogen atmosphere. The temperature was maintained at 600 °C for 2 h, after which the sample was allowed to cool. Following this, the sample was removed from the furnace, dissolved in a 2 M hydrochloric acid solution, and stirred on a magnetic stirrer for 0.5 h. The mixture was then soaked for 12 h. After soaking, the sample was washed with distilled water and dried in an oven at 80 °C for 7 h to obtain the rice straw biochar, labeled as RSBC.

**Figure 12:**
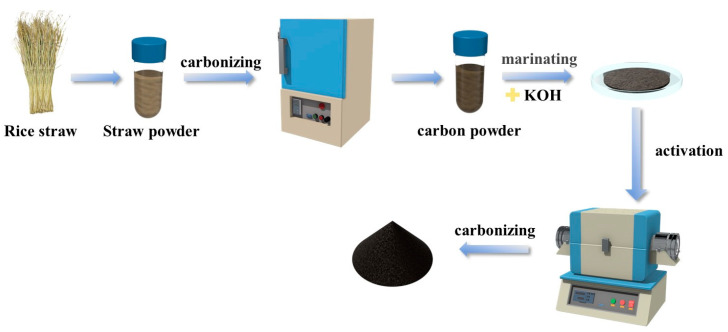
Schematic illustration of preparation of activated rice straw biochar.

The prepared rice straw biochar was mixed with KOH in a specific ratio, and distilled water was added to dissolve the mixture. The mixture was then stirred and dried for a specified period. After drying, the powder was ground to a uniform consistency and placed into a tube furnace. The temperature was gradually increased to a predetermined value at a rate of 5 °C/min under a nitrogen atmosphere, held for 2 h at that temperature, and then allowed to cool. Once cooled, the powder was added to a specific amount of 1M nitric acid solution and subjected to stirring and ultrasonic treatment to ensure complete reaction. After the reaction, the mixture was washed with deionized water and vacuum dried to obtain the final activated rice straw biochar sample. A schematic representation of the activated rice straw biochar preparation process is illustrated in Figure 12.

### 3.2. Materials Characterization

X-ray powder diffraction (XRD) analysis was performed using a Shimadzu XRD-7000 diffractometer (Shimadzu Corporation, Kyoto, Japan) with a Cu Kα radiation source. The measurement parameters included a tube voltage of 40.0 kV and a tube current of 30.0 mA. The diffraction patterns were recorded within a scanning range of 10° to 80°, with a scan rate of 6°·min^−1^. The morphology of the samples was examined using scanning electron microscopy (SEM) on a Zeiss Merlin Compact, (Zeiss, Oberkochen, Germany), with an accelerating voltage of 30 kV. Raman spectroscopy was conducted using a Horiba LabRAM HR Evolution spectrometer (Horiba Scientific, Kyoto, Japan) with a 532 nm excitation laser, and spectral data were collected within the range of 60 to 3000 cm^−1^. The specific surface area and pore size distribution of the samples were measured using a Beijing Jingwei Gaobo BET analyzer (Beijing Jingwei Gaobo Technology Co., Ltd., Beijing, China).

### 3.3. Electrochemical Measurements

The active material powder, acetylene black, and binder (PTFE) were weighed and mixed in a mass ratio of 75:20:5. An appropriate amount of isopropanol was added as a dispersant, and the mixture was ground in a mortar to form a slurry. This slurry was then applied to a foam nickel substrate and dried in a vacuum oven at 80 °C for 12 h. Both the positive and negative electrode sheets were immersed in a 6 M KOH solution for 4 h, while the separator was soaked in a separate 6 M KOH solution for the same duration.

Supercapacitors were assembled using two electrode sheets. A single electrode sheet, with the active material facing upward, was placed inside the negative shell. A separator, wetted with electrolyte, was positioned on top of the electrode. Another electrode sheet, with the active material facing downward, was carefully aligned with the first electrode. A 0.5 mm thick spacer and a 0.15 mm foam nickel sheet were then added sequentially to ensure proper alignment of all components. The positive shell was placed on top and aligned with the assembly. The coin cell was completed and pressed.

Electrochemical impedance spectroscopy (EIS) was conducted using an electrochemical workstation (CHI660C, Shanghai Chenhua Instrument Co., Ltd., Shanghai, China) with an amplitude of 5 mV and a frequency range of 0.01 Hz to 100 kHz. Cyclic voltammetry (CV) was performed with scan rates ranging from 5 to 200 mV/s. Galvanostatic charge–discharge (GCD) measurements of the supercapacitors were carried out using a LANHE CT3002A, within a voltage range of -1 to 0 V.

The specific capacitance of activated rice straw biochar was calculated based on the following equation [36]:

(1) $$C^{\prime }=\frac{4I∆t}{m^{\prime }∆V}$$

where *I* is the discharge current, *m*′ is the total mass of two electrodes, Δ*t* is the discharge time, and Δ*V* is the discharge voltage.

## 4. Conclusions

In conclusion, activated rice straw biochar (RSBC) was synthesized through thermal treatment with KOH as the activation agent under various activation conditions. The materials were characterized using X-ray diffraction (XRD), scanning electron microscopy (SEM), Raman spectroscopy, and Brunauer–Emmett–Teller (BET) analysis and their electrochemical performance as supercapacitor electrodes was evaluated. Activation significantly increased the specific surface area of the samples, which is crucial for enhancing their electrochemical properties. The optimal electrochemical performance was achieved under the conditions of an activation temperature of 800 °C, an activation ratio of 1:2, and an activation time of 0.5 h. Under these conditions, RSBC-2 exhibited a specific capacitance of 296 F g^−1^ at a current density of 0.2 A g^−1^, with a capacity retention of 81% when the current density increased from 0.2 to 5 A g^−1^. These results underscore the importance of optimizing activation parameters—such as temperature, ratio, and time—in improving the electrochemical performance of biochar. This study demonstrates the potential of utilizing rice straw as a sustainable, high-performance material for energy storage applications. It offers a promising approach for the high-value utilization of agricultural waste in energy storage, contributing to the development of environmentally friendly and efficient energy storage technologies.

## Figures and Tables

**Table 1:** The specific capacitance of rice straw biochar at different activation temperatures under various current densities.

Samples	0.2 A/g	0.5 A/g	1 A/g	2 A/g	5 A/g
RSBC-700	147.6 F g^−1^	134 F g^−1^	128 F g^−1^	124 F g^−1^	120 F g^−1^
RSBC-800	197.2 F g^−1^	185 F g^−1^	178 F g^−1^	172 F g^−1^	170 F g^−1^
RSBC-900	86.8 F g^−1^	83 F g^−1^	82 F g^−1^	80 F g^−1^	80 F g^−1^

**Table 2:** The specific capacitance of rice straw biochar with different activation ratios under various current densities.

Samples	0.2 A/g	0.5 A/g	1 A/g	2 A/g	5 A/g
RSBC-1	222 F g^−1^	209 F g^−1^	198 F g^−1^	160 F g^−1^	137 F g^−1^
RSBC-2	296 F g^−1^	281 F g^−1^	272 F g^−1^	260 F g^−1^	240 F g^−1^
RSBC-3	164 F g^−1^	158 F g^−1^	147 F g^−1^	135 F g^−1^	125 F g^−1^

**Table 3:** The specific capacitance of rice straw biochar with different activation times under various current densities.

Samples	0.2 A/g	0.5 A/g	1 A/g	2 A/g	5 A/g
RSBC-0.5h	296 F g^−1^	281 F g^−1^	272 F g^−1^	260 F g^−1^	240 F g^−1^
RSBC-6h	254 F g^−1^	239 F g^−1^	226 F g^−1^	160 F g^−1^	120 F g^−1^
RSBC-12h	232 F g^−1^	222 F g^−1^	214 F g^−1^	190 F g^−1^	180 F g^−1^

**Table 4:** Specific surface area and pore size of RSBC and RSBC-2.

Samples	S_BET_ (m^2^ g^−1^)	V_t_ (cm^3^ g^−1^)	Average Pore Size (nm)
RSBC	75.339	0.087	4.630
RSBC-2	939.404	0.533	2.268

## Data Availability

Data will be made available on request.

## Supplementary Materials

The following supporting information can be downloaded at: https://www.mdpi.com/article/10.3390/molecules30030632/s1, Figures S1 and S2: The cycling stability tests for RSBC-2; TG-DTG curves of rice straw biochar before and after activation.
