# Supplementary material for: Impact of Activation Conditions on the Electrochemical Performance of Rice Straw Biochar for Supercapacitor Electrodes
Source: Molecules. 2025 Jan 31;30(3):632. doi: 10.3390/molecules30030632 (PMC11820247; doi:10.3390/molecules30030632)
Supplement: Supplementary file 1 [file molecules-30-00632-s001.zip › molecules-3432180-supplementary.pdf]

Impact of Activation Conditions on the Electrochemical Performance of Rice Straw Biochar for Supercapacitor Electrodes

Jialuo Cheng[1,2], Yumeng Lu[3], Ya Sun[1*], Sunhua Deng[4], Heng Yang[1], Manman Zhang[1], Chunlei Wang[1], Juntao Yan[1*]

1 College of Chemistry and Environmental Engineering, Key Laboratory of Agricultural Waste Resource Utilization in Hubei Province, Wuhan Polytechnic University, Wuhan 430023, China

2 Institute of Plant Protection and Soil Fertilizer, Hubei Academy of Agricultural Sciences, Wuhan 430064, China

3 School of Nursing and Health Management, Wuhan Donghu University, Wuhan 430212, China

4 College of Construction Engineering, Jilin University, Changchun 130021, China

* Corresponding author. Email: sunya230@whpu.edu.cn (Y.S); 12111@whpu.edu.cn (J.Y)

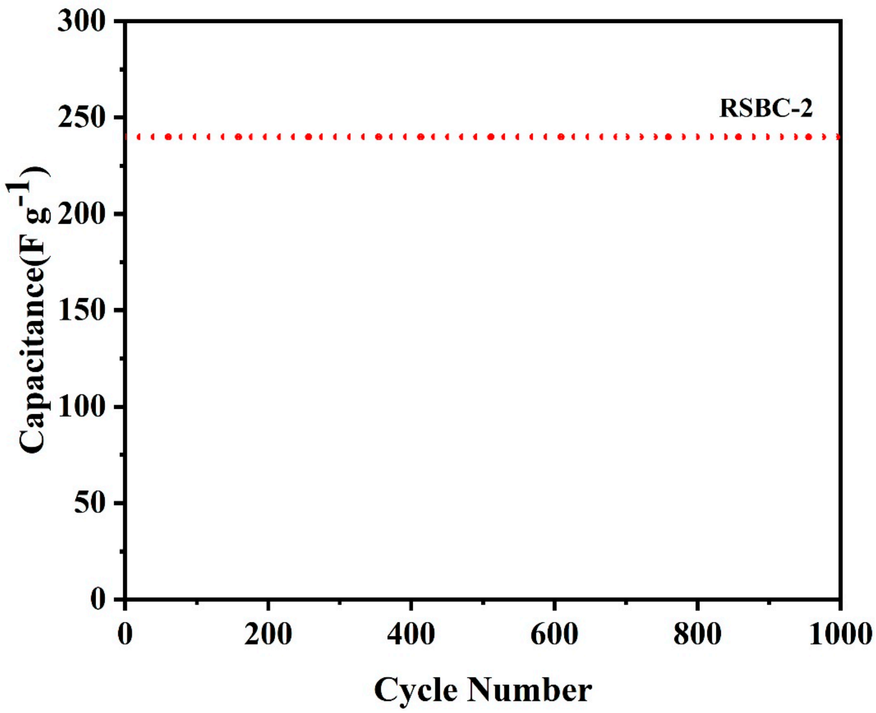

Figure S1 Cycling performance of RSBC-2 at a current density of 5 A g^{-1} for 1000 cycles.

The cycling stability tests for RSBC are shown in Figure S1. The RSBC-2 was cycled for 1000 cycles at a current density of 5 A g^{-1}. After 1000 cycles, RSBC-2 retained 100% of its capacity, indicating that the electrode materials exhibit excellent cycling stability.

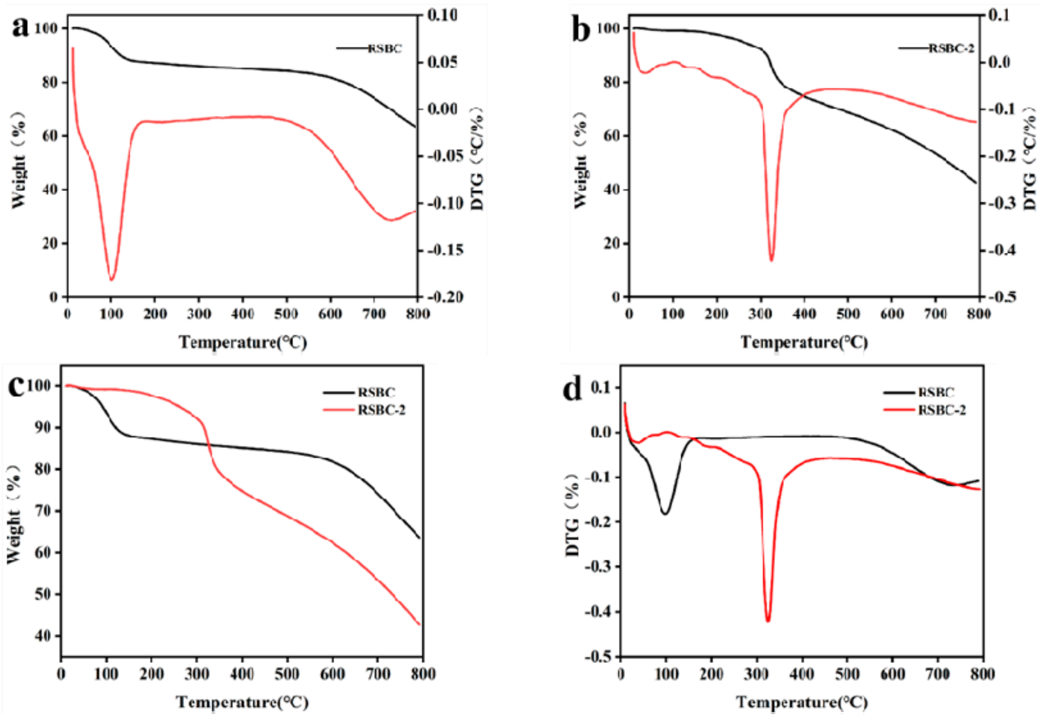

Figure S2 TG-DTG curves of rice straw biochar before and after activation.

To investigate the thermal stability of different samples, the initial sample RSBC and the best-performing RSBC-2 were subjected to thermal stability tests at a heating rate of 10°C/min in a nitrogen atmosphere. As shown in Figure S2, the TG-DTG comparison of rice straw biochar before and after activation is presented. The TG curve shows the change in sample mass with respect to temperature and time, while the DTG curve reflects the rate of mass change with respect to temperature and time. Figure S2a show the TG and DTG curves of RSBC, where the pyrolysis can be divided into three stages. The first stage occurs from 0°C to approximately 150°C, during which a mass loss of about 12% is observed, primarily due to the surface-adsorbed moisture and some volatile organic compounds. The second stage occurs from 150°C to 600°C, with a mass loss of about 20%. The third stage, from 600°C to 800°C, shows a mass loss of approximately 40%. At higher temperatures, the main components of RSBC undergo pyrolysis and mass loss, which leads to multiple peaks in mass loss. Figure S2b shows the TG and DTG curves of RSBC-2, where the mass loss is divided into two stages. The first stage occurs from 0°C to around 300°C, with a mass loss of about 10%, and the second stage occurs from

300°C to 800°C, with a mass loss of about 60%. Figures S2c -d provide a clearer observation of the pyrolysis rate and mass loss proportion of RSBC and RSBC-2. In the first stage, the higher the surface moisture content, the faster the rate of mass loss and the greater the proportion of mass loss. For energy storage materials, higher moisture content can lead to oxidation and corrosion of the electrode material, which in turn reduces its electrochemical performance.
